# µ-PIV Measurements of Flows Generated by Photolithography-Fabricated Achiral Microswimmers

**DOI:** 10.3390/mi10120865

**Published:** 2019-12-10

**Authors:** Liyuan Tan, Jamel Ali, U Kei Cheang, Xiangcheng Shi, Dalhyung Kim, Min Jun Kim

**Affiliations:** 1 Department of Mechanical and Energy Engineering, Southern University of Science and Technology, Shenzhen 518055, China; 11749160@mail.sustc.edu.cn (L.T.); x.shi@u.nus.edu (X.S.); 2 Department of Chemical and Biomedical Engineering, FAMU-FSU College of Engineering, Tallahassee, FL 32310, USA; jali@eng.famu.fsu.edu; 3 National High Magnetic Field Laboratory, 1800 East Paul Dirac Drive, Tallahassee, FL 32310, USA; 4 Department of Mechanical Engineering, Kennesaw State University, Marietta, GA 30060, USA; dkim97@kennesaw.edu; 5 Department of Mechanical Engineering, Southern Methodist University, Dallas, TX 75275, USA; mjkim@lyle.smu.edu

**Keywords:** microrobotics, magnetic control, low Reynolds number

## Abstract

Robotic micro/nanoswimmers can potentially be used as tools for medical applications, such as drug delivery and noninvasive surgery. Recently, achiral microswimmers have gained significant attention because of their simple structures, which enables high-throughput fabrication and size scalability. Here, microparticle image velocimetry (µ-PIV) was used to study the hydrodynamics of achiral microswimmers near a boundary. The structures of these microswimmers resemble the letter L and were fabricated using photolithography and thin-film deposition. Through µ-PIV measurements, the velocity flow fields of the microswimmers rotating at different frequencies were observed. The results herein yield an understanding of the hydrodynamics of the L-shaped microswimmers, which will be useful in applications such as fluidic manipulation.

## 1. Introduction

Micro/nanoswimmers have been intensely investigated for the past decade because of their potential applications in drug delivery [1,2], biological sensing [3,4], and tissue manipulation [5,6]. At the microscale, these devices swim at low Reynolds numbers, where viscous forces dominate over inertia; thus, the fluid flow becomes time reversible. According to the scallop theorem [7], nonreciprocal motion is required to achieve a net forward thrust at low Reynolds numbers. To generate nonreciprocal swimming strokes in viscosity-dominated environments, many existing micro/nanoswimmers utilize helical structures or flexible bodies.

Rigid helical micro/nanoswimmers mimic the swimming motion of bacteria such as *Escherichia coli* and can be obtained using a number of top-down and bottom-up techniques, including self-scrolling [8], 3D direct laser writing [9], and nucleic acid manipulation [10]. Microswimmers with flexible bodies are analogous to the flagella of sperm, which can generate propulsion by propagating nonreciprocal traveling waves down the flexible flagellum [11]. So far, most of the flexible microswimmers have been constructed with rigid segments that are connected by soft junctions. However, the complex or costly fabrication process of these two kinds of microswimmers limits their further application, despite their swimming properties [12]. Chemically driven propulsion with hydrogen peroxide (H_2_O_2_) has the fastest swimming speed among microswimmers thus far, but this requires specific chemical environments for actuation [13,14]. Other systems such as acoustic- [15], light- [16], and electrostatic-activated [17] microswimmers have also attracted significant attention; here, we focused on magnetically driven propellers.

Apart from helical or flexible body microswimmers, studies have demonstrated that rigid achiral microswimmers can also swim at low Reynolds numbers under rotating magnetic fields, such as particle-based microswimmers [18], which was the first reported achiral microswimmer, in part to re-examine the minimal geometrical requirements for designing microswimmers. In a follow-up study, Cheang and Kim [19] thoroughly discussed the feasibility of fabricating achiral microswimmers that, because of their 2D simplistic geometries, could be fabricated at low cost using high-throughput lithography techniques. This was later corroborated in experiments with planar microswimmers [20]. Furthermore, the swimming properties of the achiral microswimmers were extensively investigated, with the conclusion that achiral planar shapes are nearly optimal propellers [21]. Similar achiral structures with asymmetric arms have also been used to obtain imbalanced forces and induced torques in other systems [22,23].

Aside from the above-mentioned microswimmers that are designed for swimming in bulk fluid, magnetically actuated rolling microrobots are actively being studied because of their simplicity. For example, a dumbbell-like microrobot can be used for cargo transportation using the microvortices it generates [24]. Similarly, the microvortices generated by the rotational microparticles have been used for trapping objects such as live bacteria [25]. These two examples of cargo transportation using rolling robots, which took advantage of microvortices for localized fluid trapping, illustrate the importance and potential applications of studying the low Reynolds number hydrodynamics of microswimmers. However, unlike microswimmers, these devices are limited to locomotion on a surface [15,26].

Unlike their chiral counterparts (e.g. rigid helices), achiral microswimmers have not been thoroughly investigated, specifically their hydrodynamic properties are largely unknown. Understanding the hydrodynamics of a microswimmer can lead to better control and practical applications. Even though numerical simulations were conducted to study the hydrodynamics of achiral microswimmers in bulk fluid [27,28] and near boundaries [29], so far there have been no experimental reports on the flows produced by these swimmers.

The microparticle image velocimetry (µ-PIV) technique can be used to measure the velocity flow field of a microswimmer by measuring the moving patterns of laser-excited tracer particles within a fluid in sequential frames. For the past few years, µ-PIV had been used to study the flows of a number of different microswimmers. For example, µ-PIV was used to further the understanding of the swimming behavior of microorganisms [30,31,32], as well as a microorganism-based microswimmer [33], while stereoscopic µ-PIV measurements were used to confirm the asymmetric dynamic motion of artificial cilia that can generate 3D asymmetric dipole vortices [34]. For helical microswimmers, [35,36] studied the thrust force and efficiency of helical microswimmers using µ-PIV with both micro- and macroscale models. Recently, Mart´ınez-Aranda et al. studied the flow dynamics around several microswimmers prototypes, such as spherical, elliptical, and cylindrical shapes, in non-Newtonian fluids using µ-PIV in order to simulate the hydrodynamics of microswimmers inside a human vessel [37]. Moreover, a µ-PIV study was conducted to investigate the fluid flow generated by microspheres, which are widely used for micromixing and pumping, mask-free colloidal patterning, and as microrobots in the form of rolling robots [13,38]. Even though the µ-PIV technique has been used for investigating chiral and flexible microswimmers, there is still a lack of literature on achiral microswimmers.

Here we report on the µ-PIV characterization of achiral L-shaped microswimmers fabricated using photolithography and thin-film deposition, which allowed us to visualize the flow field and obtain quantitative data for validation. The results presented herein will provide a better understanding of the hydrodynamics of achiral shapes, which will aid in the future application of achiral microswimmers for microfluidic tasks.

## 2. Materials and Methods 

### 2.1. Microswimmer Fabrication

**Figure 1:**
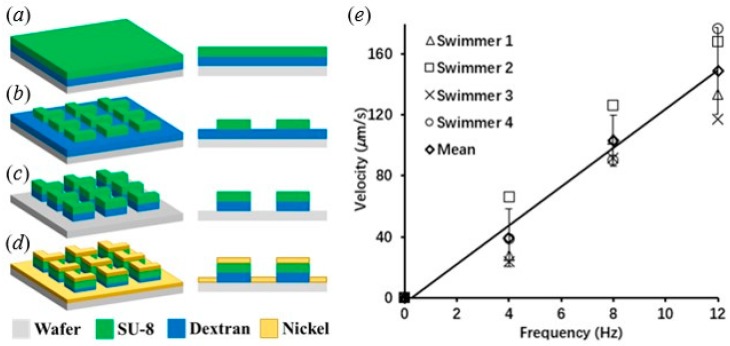
(**a**)–(**d**) Fabrication process of L-shaped microswimmers; (**e**) velocity versus rotating frequency of microswimmers.

L-shaped microswimmers were fabricated using a traditional photolithographic lift-off process as shown in Figure 1a–d. First, a clean silicon wafer was coated with a layer of dextran (5% (*w*/*v*); 60 kDa), followed by deposition of a layer of negative-tone photoresist (SU-8, Micro-Chem, MA, USA) via spin-coating. Next, a dark field transparency in contact with the photoresist was used to transfer patterns through UV exposure, followed by post-baking and development in propylene glycol monomethyl ether acetate (PGMEA). To impart ferromagnetic properties to the microstructures, a 200 nm thin film of nickel was deposited onto the substrate using thermal evaporation. Nickel pellets (99.995%, Kurt J. Lesker, PA, USA) were evaporated at a rate of 0.5 Å/s with a chamber pressure of 10^−6^ Torr. The fabricated L-shape microswimmers had an arm width, length, and thickness of 40, 120, and 2 µm, respectively. The magnetic moments of the fabricated microswimmers were aligned with the easy axis [20]. Finally, the nickel coated L-shaped structures were then released from the water-soluble sacrificial dextran layer in deionized water.

### 2.2. Magnetic Actuation and µ-PIV

**Figure 2:**
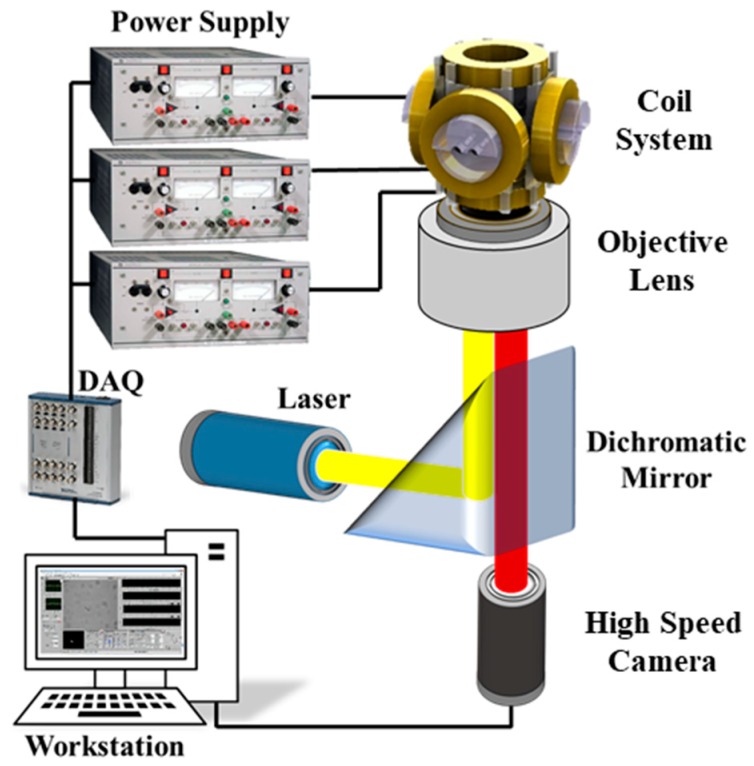
Schematic of the µ-PIV (micro-particle image velocimetry) system. DAQ, data acquisition device.

The swimming mechanism of the achiral microswimmer was studied by Sachs et al. [39] and Cheang et al. [18], and a brief analysis was provided in supplementary materials. In order to observe microswimmers in motion, we mounted an electromagnetic coil system onto a fluorescence microscope in which the PIV experiment was performed. As shown in Figure 2, the coil system was powered by three power supplies (Kepco BOP20-5M) and controlled by a National Instrument (NI) Data Acquisition (DAQ) device (PCI-6259) and a LabVIEW interface. Flow velocity measurements were obtained using previously reported methods [33,38]. The PIV experiments were conducted in water at room temperature (~1 cP). Fluorescent seeding particles (200 nm) were introduced into samples and excited using a continuous wave laser, as shown in Figure 2. Particle image sequences were acquired at 500 frames per second and subsequently analyzed. Recorded images were imported into the commercial software package (DaVis 8.0), which was used for flow field reconstruction. Instantaneous flow fields were obtained using interrogation windows measuring 32 × 32 pixels, which had an overlap of 50%. Smoothing (3 × 3) was applied to the post-processing of the obtained flow fields data. The microswimmers used for µ-PIV measurement were the same as those used for swimming velocity testing.

## 3. Results and Discussion

### 3.1. Swimming Profile

**Figure 3:**
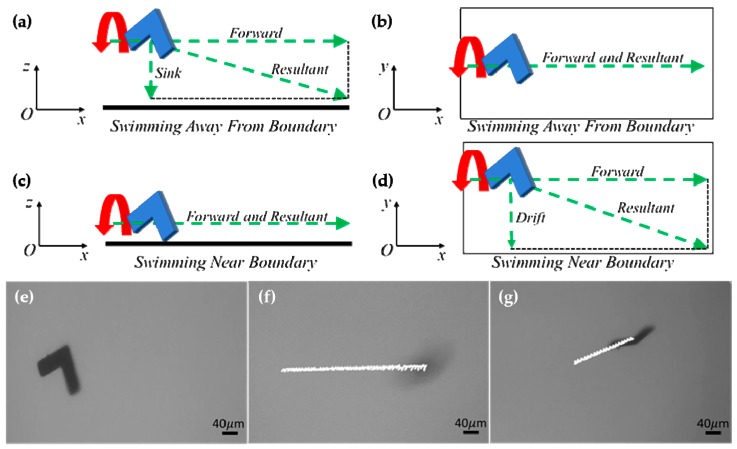
Swimming behavior in bulk fluid and near an underlying boundary. (**a**)–(**d**) Illustration of translational and drift velocity of microswimmers translating at a distance far away from the boundary and near the boundary; (**a**) sideview and velocity decomposition of a microswimmer swimming at a distance away from the boundary; (**b**) top view of a microswimmer swimming away from the boundary; (**c**) sideview of a microswimmer swimming near the boundary; (**d**) top view of a microswimmer swimming near the boundary and velocity decomposition. (**e**)–(**g**) Swimming control experiment of an L-shaped microswimmer; (**e**) L-shaped microswimmer floating in bulk fluid; (**f**) swimming of an L-shaped microswimmer in bulk fluid, at a distance far away from the boundary; (**g**) swimming of an L-shaped microswimmer near the boundary.

After fabrication, the swimming performance of the L-shaped microswimmers was evaluated under different frequencies of a rotational magnetic field. Four microswimmers of similar size were used, and each microswimmer was tested under different frequencies. As can be seen in Figure 1e, the velocity increases with the field frequency, which corroborates the results obtained by Tottori and Nelson [20]. The peak velocity of the L-shaped microswimmers was 149 µm/s on average when the frequency was increased to 12 Hz. The velocity was obtained using a tracking algorithm programmed in MATLAB. The standard variation in Figure 1e was 28.3 µm/s. The variation observed may be attributed to the magnetic moment variance that could have been introduced during pre-magnetization; this can lead to slightly different swimming motions among the four swimmers. Since the experiments were conducted with swimmers close to the boundary, a strong drifting velocity (motion perpendicular to the desired direction of swimming) was observed. A graphical illustration of drift velocity is shown in Figure 3d, in which drift velocity is along the *y*-direction. 

The motion of a microswimmer translating near the bottom surface exhibited both translational and drift swimming motion, as illustrated in Figure 3a–d, due to hydrodynamic interactions with the wall (glass slide). When the microswimmer was actuated far from the boundary (i.e., swimming at a distance from the boundary where hydrodynamic interactions with the boundary do not influence swimming behavior), it swam forward without drifting laterally. However, the microswimmer slowly sank to the bottom over time. When the microswimmer sank down to the boundary, strong drifting motion was observed. To verify whether the translational motion of a microswimmer swimming in bulk fluid (far from boundary) was the same as that of a microswimmer near a boundary, we measured the velocity of a microswimmer that was swimming in bulk fluid and near the boundary, respectively. The achiral microswimmers used here had an arm width and length of 40 and 120 µm, respectively, and with a thickness of 2 µm. Figure 3e (Video S1) shows the microswimmer in bulk fluid without applying magnetic torque. The time span of the trajectory in Figure 3f (Video S1), which shows translational motion, was 15 s with an average swimming velocity of 32.24 µm/s and a standard deviation of 5.40 µm/s. The maximum and minimum velocities during this time period were 41.14 and 24.55 µm/s, respectively. In Figure 3g (Video S1), the same microswimmer was swimming close to the surface; a strong drifting motion was observed during that 6 s. The resultant trajectories of the swimmers were decomposed into *x* and *y* coordinate velocities; movement in the *x*-direction here corresponds to translational velocity, while movement in *y* corresponds to a drift velocity induced by near-wall interactions. A mean translational swimming velocity was calculated to be 30.62 µm/s with a standard deviation of 0.82 µm/s, while 31.57 and 29.74 µm/s were the maximum and minimum velocities, respectively. The average, maximum, and minimum translational swimming velocities for both cases were in close agreement. 

### 3.2. Near-Swimmer Flow Displacement

**Figure 4:**
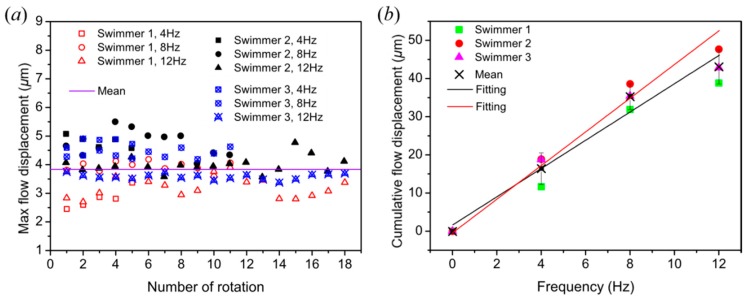
(**a**) Maximum flow displacement generated by each number of rotations; (**b**) cumulative flow displacements with different rotational frequencies. The black line is linear fit based on four average data points from 0 to 12 Hz; the red line is linear fit based on three average data points from 0 to 8 Hz.

µ-PIV measurements were conducted with three microswimmers swimming near the boundary, and the velocity flow fields in accordance with the translational direction were extracted. It can be seen in Figure 4a that the maximum flow displacement generated by each rotation was similar at different frequencies with an average of 3.83 µm. This is expected, because the displacement for one rotation should always be the same regardless of rotating frequency, thus quantitatively verifying the consistency across PIV data for different swimmers and frequencies. However, there was a discrepancy that the maximum displacement generated at the frequency of 12 Hz was smaller and mostly beneath the mean value when compared to the maximum displacement of lower frequencies, as shown in Figure 4a. The number of frames per rotation for 4, 8, and 12 Hz were 125, 63, and 42, respectively. Since the number of frames for every rotation decreased with frequencies, the details for each rotation decreased as well. As a result, a small discrepancy in the data occurred, leading to a smaller maximum displacement for 12 Hz. The discrepancy was consistent across experiments and did not have a significant impact on the results. 

The cumulative displacements at 12 and 8 Hz were three and two times that of the value at 4 Hz, respectively, which suggests a linear dependence of the flow displacement with respect to rotating frequency, as shown from the black linear fitting line with a *R*^2^ value of 0.963 from Figure 4b. This was because the cumulative maximum displacements for 8 and 12 Hz accumulated, respectively, two and three times as fast as that for 4 Hz because of the doubled and tripled angular velocity, respectively. A discrepancy occurred at 12 Hz because of the reason mentioned in the previous paragraph for Figure 4a. The cumulative displacements at 12 Hz was 43 µm, which was smaller than the expected displacements of 51 µm, according to the linear relationship. Without the discrepancy, a more linear fit, likely similar to the red linear fitting line with a *R*^2^ value of 0.996, which fit the data points from 0 to 8 Hz in Figure 4b, can be expected. The red linear fitting line extends to 12 Hz, which can be interpreted as the expected average value for 12 Hz without the discrepancy. It should also be noted that the cumulative flow displacement corresponded to the flow displacement in one second, which represents velocity.

### 3.3. Flow Field Analysis

**Figure 5:**
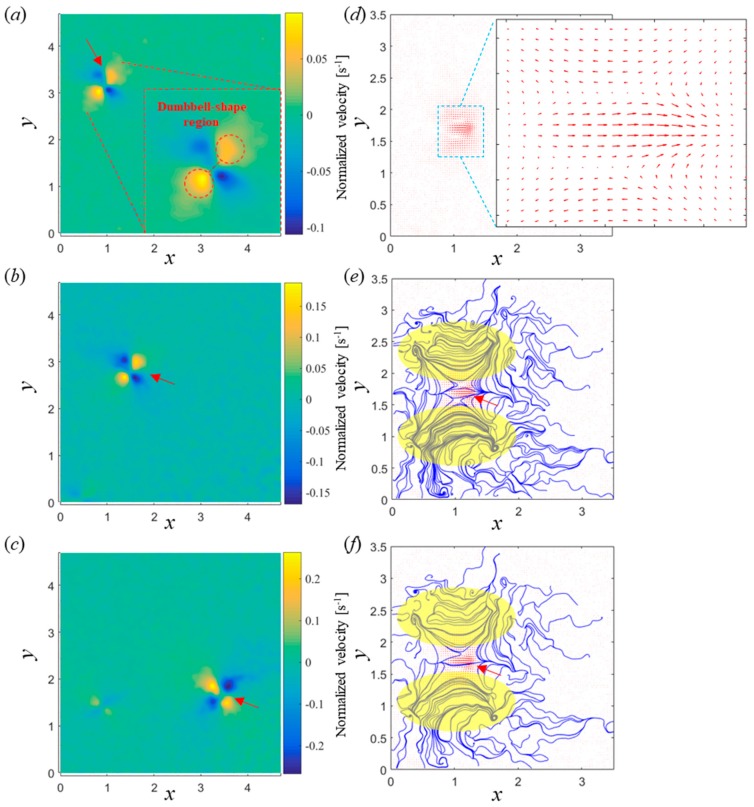
Flow fields, normalized by swimmer length, generated by achiral microswimmers at different rotating frequencies. Velocity along the *y*-direction was extracted. Displacement flow field at (**a**) 4, (**b**) 8, and (**c**) 12 Hz. The dumbbell-shaped region in (a)–(c), shown in inset of (a), show the local flow produced around the swimmer. (**d**) Velocity vector map generated by an achiral microswimmer rotating at 4 Hz; (**e**) intensive streamlines around the rotating microswimmer; (**f**) sparse streamlines around the rotating microswimmer. The yellow elliptic regions in (**e**), (**f**) labeled the microvortices generated by the microswimmer. The red arrows in (**a**)–(**c**), (**e**), and (**f**) identify the position of the microswimmer.

As shown in Figure 5a–c, the flow patterns were very similar at different rotation frequencies as expected. The color map represents the strength of flow velocity with max flow velocity increasing linearly, corresponding to the results in Figure 4b. Four distinct flow regions of similar magnitude were observed around the microswimmer; with opposite regions having flow displacements of the same sign and forming a dumbbell shape, as shown in the inset of Figure 5a with red dash circles. The velocity vector map and streamlines of a rotating L-shaped microswimmer at 4 Hz is presented in Figure 5d–f. As shown in Figure 5e,f, two microvortices, labeled by translucent yellow elliptic regions, can be observed. With those microvortices, it might be possible to use these rotating microswimmers to conduct noncontact manipulation, such as particle trapping and transport, as demonstrated previously by Zhou et al. (2017). Limited by the nature of µ-PIV, only 2D side views of the microvortices were observed; it is speculated that the 2D flow patterns in the yellow region represent cone-shaped vortices in 3D. The intensive streamlines in Figure 5e at the yellow regions suggest a relatively stronger flow field of the microvortices than in the background. The flow field with sparse streamlines in Figure 5f clearly showed the streamlines going back and forth and illustrates the planar movement of trapped particles. Moreover, as shown in Figure 5e,f, the flow field of the rotating microswimmer covered an area of 400 × 200 µm^2^, while the microswimmer had a body length of 170 µm; therefore, this can be used to determine hydrodynamic interactions between microswimmers for relevant applications, such as swarm control. Flow field patterns of different microswimmers are provided in Supplementary Materials (Figures S1–S6).

**Figure 6:**
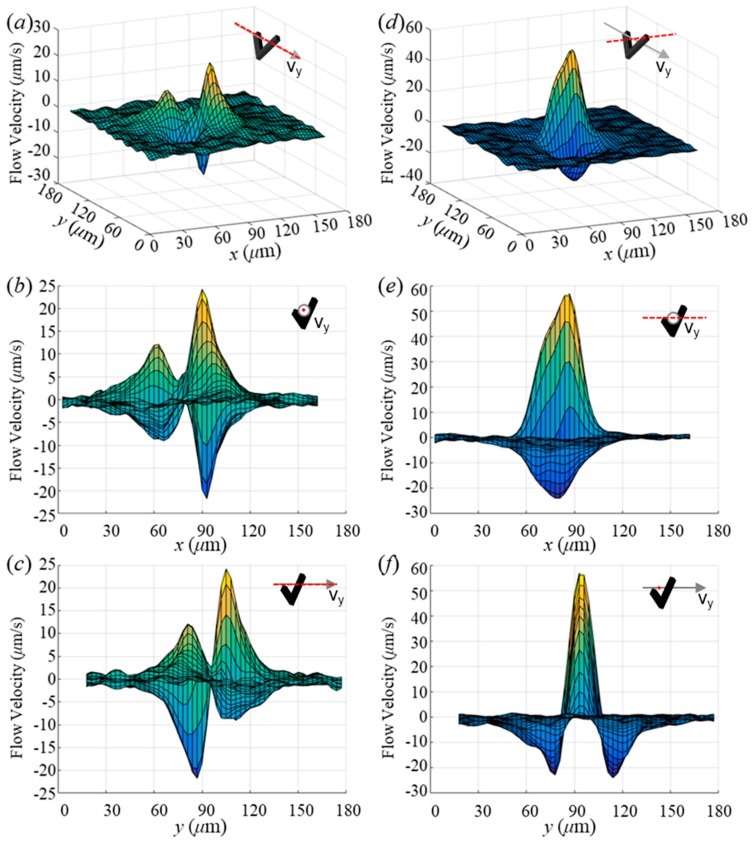
Flow field decay along *x* and *y* directions. (**a**)–(**c**) Flow velocity along the desired swimming direction; (**d**)–(**f**) flow velocity along the direction perpendicular to desired swimming direction; (**a**), (**d**) angled view of velocity profile; (**b**), (**e**) front view of velocity profile; (**c**), (**f**) left view of velocity profile. The inset in each plot is a schematic of the swimmer where the red dash line represents the datum line that was used to obtain velocities.

Flow velocity data obtained from datum lines along directions parallel and perpendicular to the desired swimming direction were plotted (Figure 6). As shown in Figure 6a–c, the flow velocity magnitude along the desired swimming direction was associated with two sets of similar dumbbell-shaped regions; however, the velocity magnitudes of one of these regions was approximately twice that of the other. The region of lower peak velocity is variant because of the different swimming motions between microswimmers, due to the same reason that led to velocity deviation in Figure 1e, while the higher peak was similar, as shown in Figure 6a, Figures S5a and S6a. Flow velocity decay profiles of different microswimmers of various frequencies are displayed in Figures S3 to S6. As one can see, all microswimmers exhibited a similar velocity decay profile. Moreover, the peaks occurred close to the middle of the microswimmer; that is, the peak velocity exponentially decayed in the radial direction from the center of the swimmer. Flow velocity along the perpendicular direction was observed to be larger than that along the direction of swimming because of the strong drift motion, as depicted in Figure 6d–f. This also explains the apparent flow along the *x*-direction shown in the inset of Figure 5d. Furthermore, a stagnation point in Figure 6e can be found close to the center of the flow field pattern along the direction of swimming, while two sets of stagnation points exist for the perpendicular direction as lower flow velocity appeared at larger distances (40 µm), as shown in Figure 6f. 

## 4. Conclusions

In this paper, the hydrodynamics of swimming L-shaped microswimmers were investigated using µ-PIV. L-shaped microswimmers were fabricated using photolithography combined with physical vapor deposition, which imparted magnetic properties onto the L-shaped structures. Velocity profiles were investigated at different frequencies and showed a swimming velocity of two body lengths per second (140 µm/s) at 12 Hz. Before conducting the µ-PIV experiment, the isolation of the translational velocity component from the drifting velocity was validated. For the µ-PIV results, the maximum displacement of the flow field generated by the microswimmers at different frequencies for each rotation were in close agreement. As expected, the cumulative displacement for each second increased with the rotational frequency increasing and showed a linear relationship. Two microvortices were found around the rotating microswimmer, covering an area of 400 × 200 µm^2^. Finally, the velocity decay profiles along both the desired swimming direction and the direction perpendicular to swimming were analyzed. This information will be useful in future investigations aimed at elucidating the hydrodynamic interaction between swimmers. Further, based on these findings, it is conceivable to utilize the characteristics of the microswimmers flow field to perform hydrodynamic manipulation, such as noncontact manipulation and transport of micro-objects through hydrodynamic trapping. Moreover, visualization of the flow field will enable the successful implementation of applications that requires swimmer-to-swimmer interactions, such as swarm control.

## Supplementary Materials

The following are available online at https://www.mdpi.com/2072-666X/10/12/865/s1. Video S1: Swimming near and away from a planar surface, Figure S1: Magnetic field profile generated by approximate Helmholtz coil, Figure S2: Flow field generated by two swimmers under different actuating frequencies, Figure S3: Flow field velocity decay profile of the swimmer in Figure 6 under 8 Hz actuation, Figure S4: Flow field velocity decay profile of the swimmer in Figure 6 under 12 Hz actuation, Figure S5: Flow field velocity decay profile of swimmer 2 under 8 Hz actuation, Figure S6: Flow field velocity decay profile of swimmer 3 under 8 Hz actuation.
