# Supplementary material for: µ-PIV Measurements of Flows Generated by Photolithography-Fabricated Achiral Microswimmers
Source: Micromachines (Basel). 2019 Dec 10;10(12):865. doi: 10.3390/mi10120865 (PMC6953064; doi:10.3390/mi10120865)
Supplement: Supplementary file 1 [file micromachines-10-00865-s001.zip › micromachines-656296-supplementary-cced.docx]

Supplementary Materials: µ-PIV Measurements of Flows Generated by Photolithography-Fabricated Achiral Microswimmers

Liyuan Tan, Jamel Ali, U Kei Cheang, Xiangcheng Shi, Dalhyung Kim and Min Jun Kim

1. Swimming mechanism of achiral microswimmers

The propulsion of the achiral swimmers can be described using the coupled rotational and translational motion of the achiral swimmer:

| $\left[ \begin{matrix} \boldsymbol{v} \\ \boldsymbol{\Omega} \end{matrix} \right]=\left[ \begin{matrix} \boldsymbol{A} & \boldsymbol{B} \\ \boldsymbol{B}^{T} & \boldsymbol{C} \end{matrix} \right] \left[ \begin{matrix} \boldsymbol{F} \\ \boldsymbol{\tau} \end{matrix} \right]$ | (1) |
| --- | --- |

where ***A***, ***B***, and ***C*** relate the translational velocity and force, translational velocity and torque, and rotational velocity and torque respectively. Due to the uniform field generated from the coil system, there is no net external force; thus, $\boldsymbol{\tau} = \boldsymbol{m}\times\boldsymbol{H}$ and $\boldsymbol{F=0}$. Therefore, $\boldsymbol{v} =\boldsymbol{B}\cdot\boldsymbol{\tau}=\boldsymbol{B}\cdot\left( \boldsymbol{m}\times\boldsymbol{H} \right)$ and a nonzero $\boldsymbol{B}$ would lead to a nonzero translational velocity $\boldsymbol{v}$. A nonzero $\boldsymbol{B}$ of this L-shaped structure could be obtained by calculation [1]. Tottori *et al*. also stated that this kind of L-shaped structures have a nonzero $\boldsymbol{B}$ [2].

2. Magnetic field generated by control system

To ensure the translational motion of microswimmers was caused only by converting rotational motion into translational motion, rather than by the pulling force of a magnetic gradient, an electromagnetic coil system was arranged in an approximate Helmholtz configuration [3] which exerts an external torque on the microswimmers through a uniform magnetic field (see Figure S1).

Helmholtz coil systems have been widely adopted to generate net torque on magnetic microswimmers [4,5]. Helmholtz coils have been accepted as a tool to generate uniform magnetic field in a localized area; this has been thoroughly validated in previous studies through experiments and simulations [6–8]. Moreover, the field strength can be calculated using the Biot-Savart law. As for the approximate Helmholtz coil that we used in this study, the field strength was tested and simulated which showed an approximately uniform region of 2 × 2 mm. Since the microswimmers were micron-sized, the 2 × 2 mm uniform area was large enough for the *μ*-PIV measurement. To show this, we use the Biot-Savart law

| $B=\frac{\mu_{0}IR^{2}}{2\left( R^{2}+\left( x+s \right)^{2} \right)^{3/2}}+\frac{\mu_{0}IR^{2}}{2\left( R^{2}+\left( x-s \right)^{2} \right)^{3/2}}$ | (2) |
| --- | --- |

to calculate the field profile of a pair of coils using MATLAB. The result is shown in the Figure S1 below.


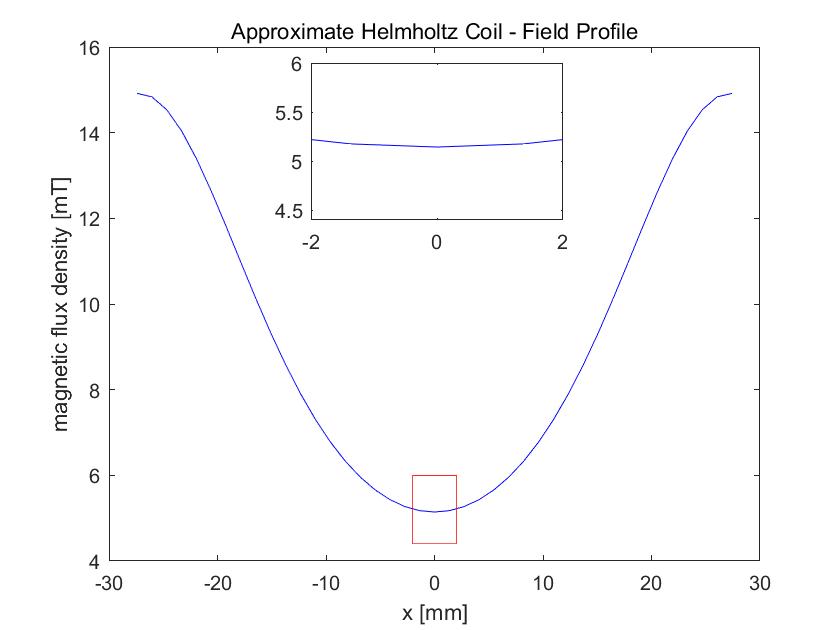


**Figure S1.** Magnetic field profile generated by approximate Helmholtz coil.

3. Flow field

**
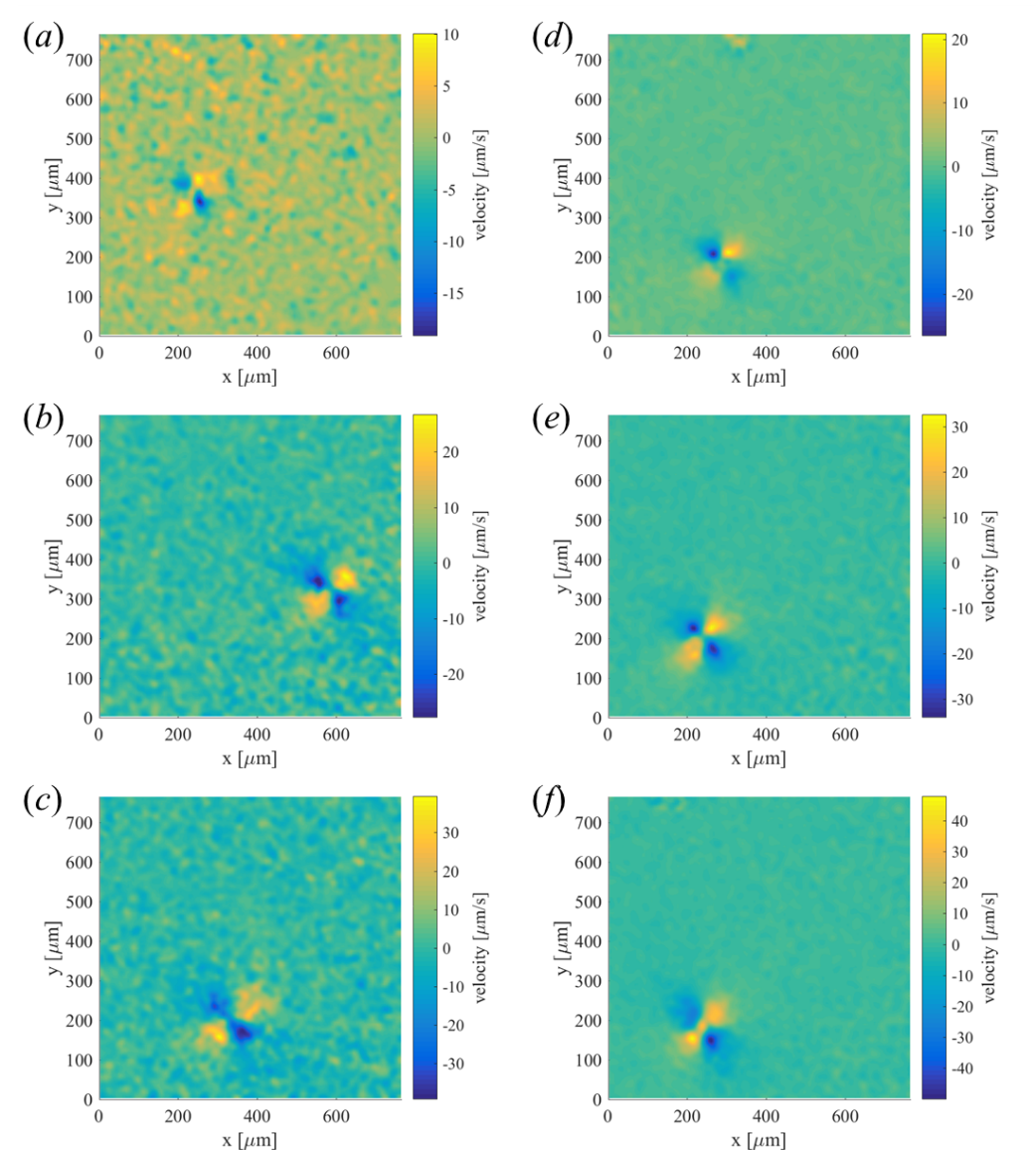
**

**Figure S2.** Flow field generated by two swimmers under different actuating frequencies. (**a**)–(**c**) Flow field generated by swimmer 2 under 4 Hz, 8 Hz and 12 Hz, respectively; (**d**)–(**f**) Flow field generated by swimmer 3 under 4 Hz, 8 Hz and 12 Hz, respectively.


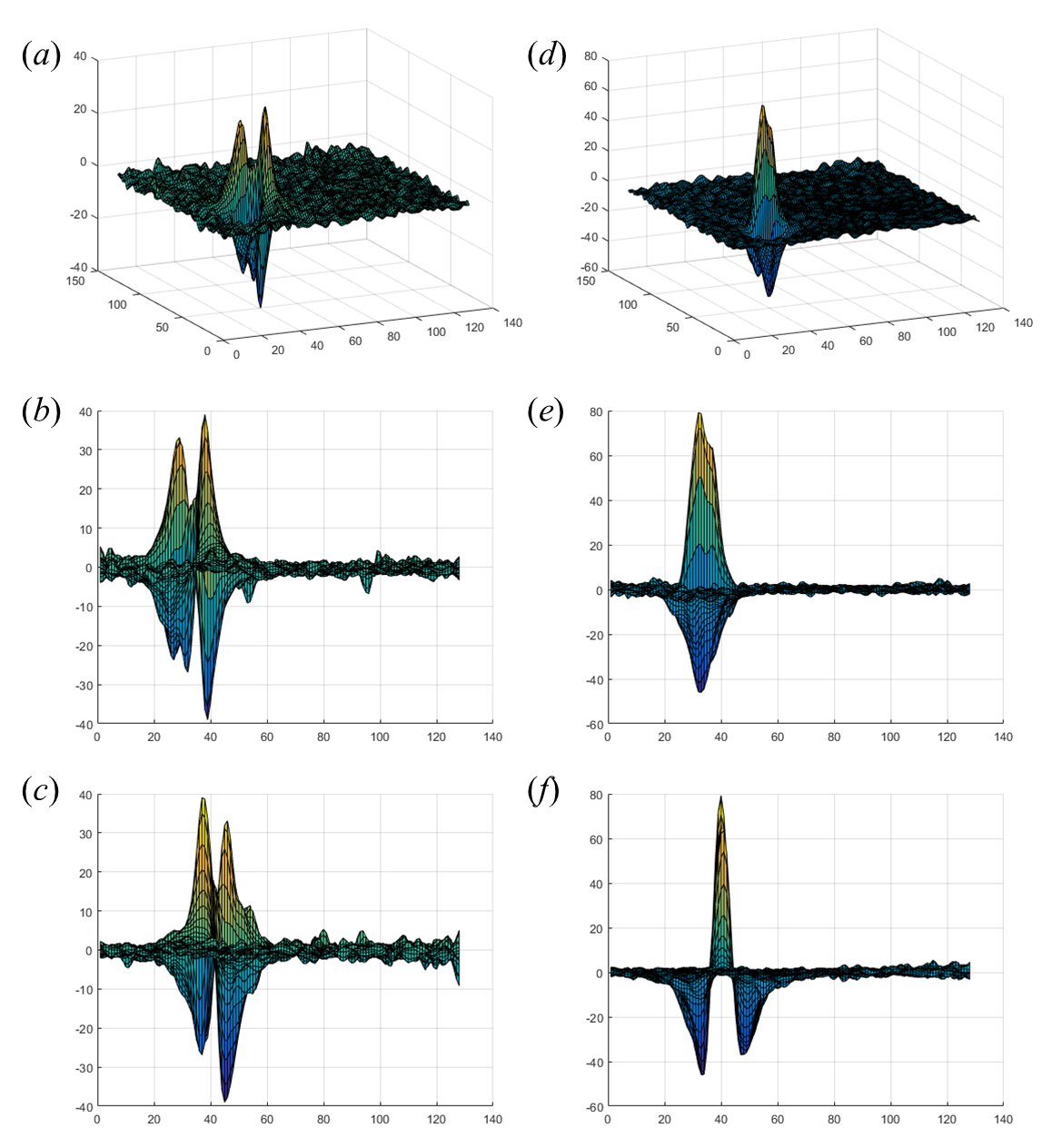


**Figure S3.** Flow field velocity decay profile of the swimmer in Figure 6 under 8 Hz actuation. (**a**)–(**c**) flow velocity along the desired swimming direction; (**d**)–(**f**) flow velocity along the direction perpendicular to desired swimming direction; (**a**), (**d**) angled view of velocity profile; (**b**), (**e**) front view of velocity profile; (**c**), (**f**) left view of velocity profile.


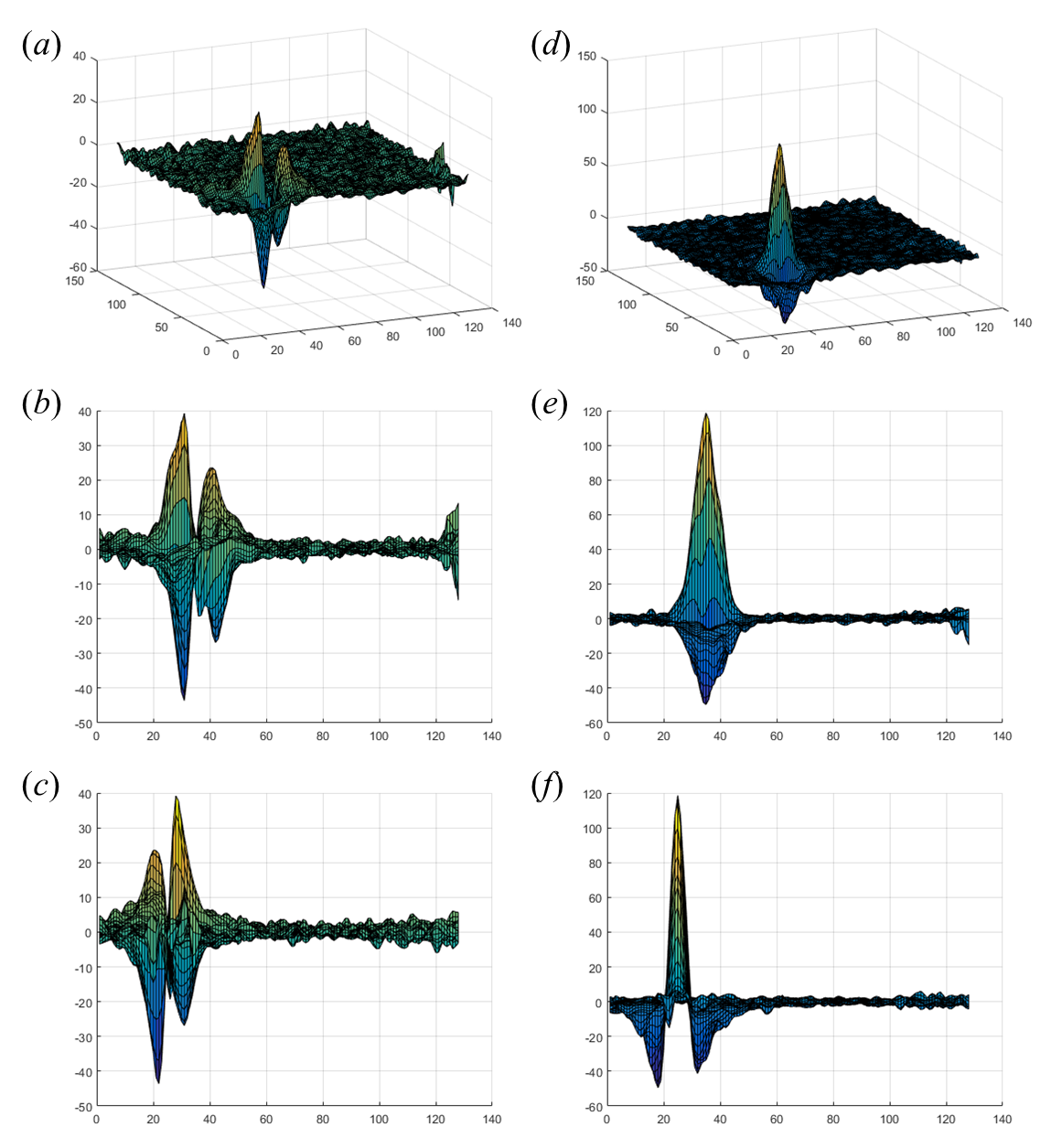


**Figure S4.** Flow field velocity decay profile of the swimmer in Figure 6 under 12 Hz actuation. (**a**)–(**c**) flow velocity along the desired swimming direction; (**d**)–(**f**) flow velocity along the direction perpendicular to desired swimming direction; (**a**), (**d**) angled view of velocity profile; (**b**), (**e**) front view of velocity profile; (**c**), (**f**) left view of velocity profile.


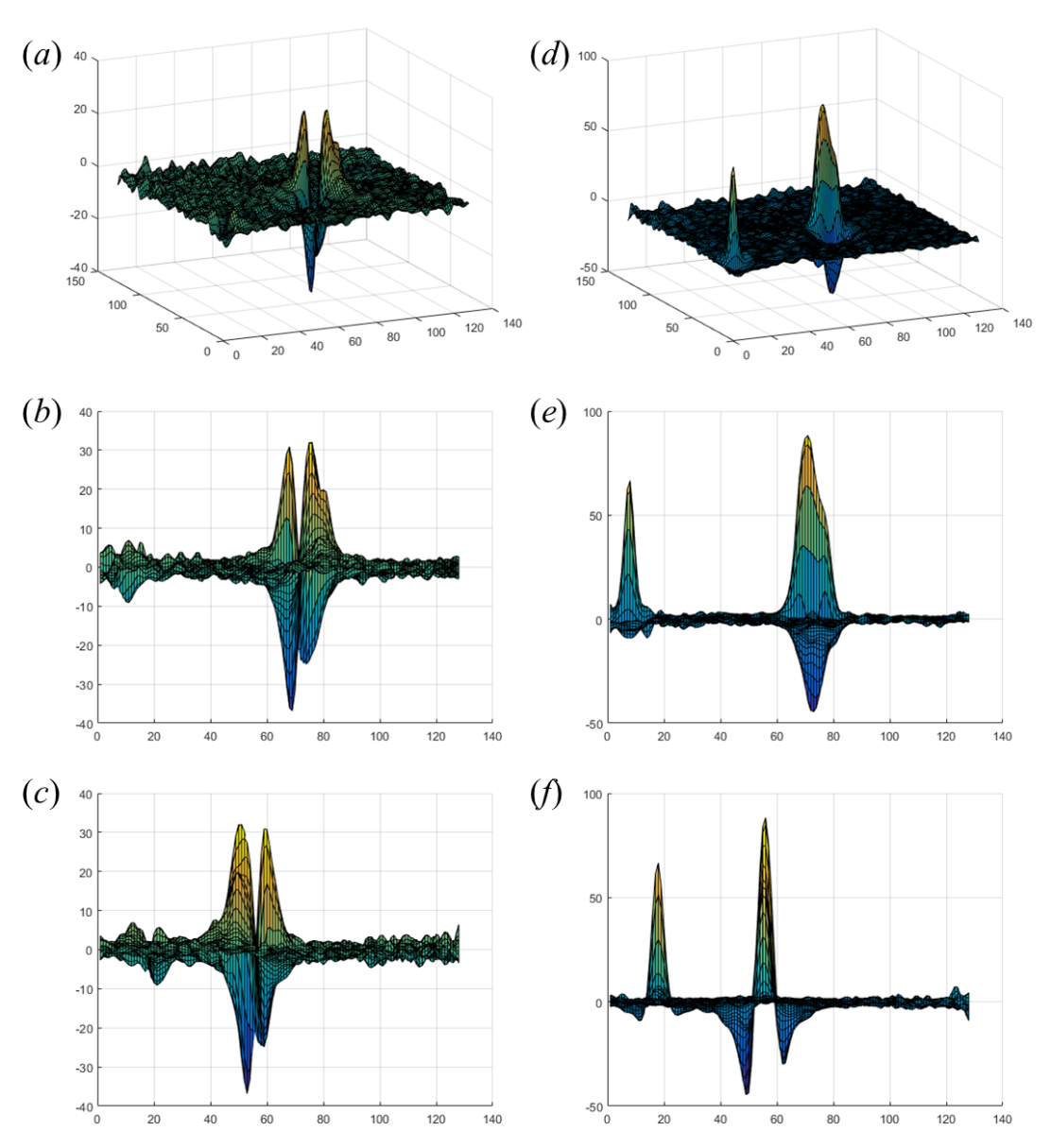


**Figure S5.** Flow field velocity decay profile of swimmer 2 under 8 Hz actuation. (**a**)–(**c**) flow velocity along the desired swimming direction; (**d**)–(**f**) flow velocity along the direction perpendicular to desired swimming direction; (**a**), (**d**) angled view of velocity profile; (**b**), (**e**) front view of velocity profile; (**c**), (**f**) left view of velocity profile.


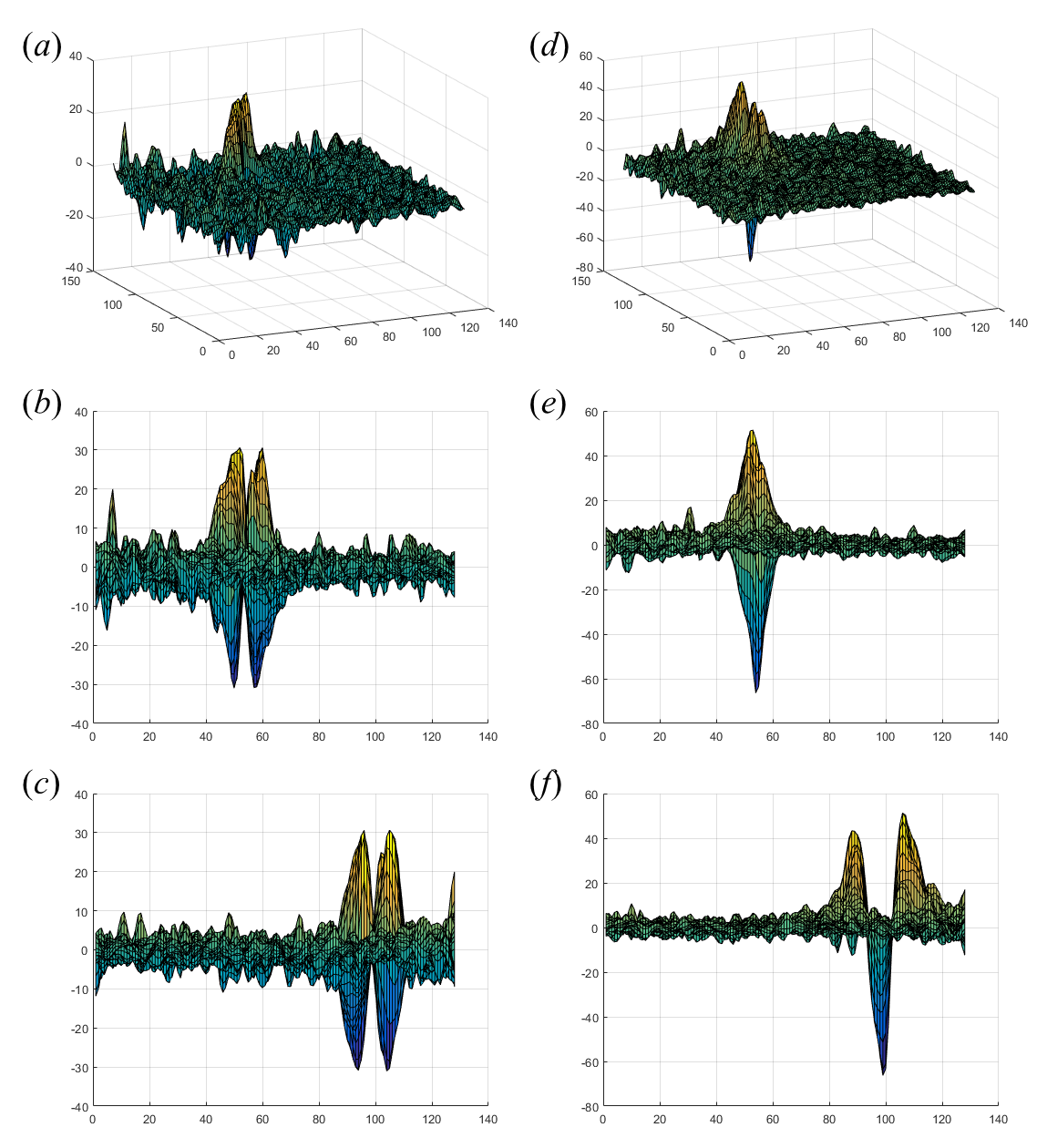


**Figure S6*.*** Flow field velocity decay profile of swimmer 3 under 8 Hz actuation. (**a**)–(**c**) flow velocity along the desired swimming direction; (**d**)–(**f**) flow velocity along the direction perpendicular to desired swimming direction; (**a**), (**d**) angled view of velocity profile; (**b**), (**e**) front view of velocity profile; (**c**), (**f**) left view of velocity profile.

1. Happel J, Brenner H. *Low Reynolds number hydrodynamics: with special applications to particulate media*; Springer Science & Business Media: Berlin/Heidelberg, Germany, 2012.
2. Tottori S, Nelson BJ. Controlled Propulsion of Two‐Dimensional Microswimmers in a Precessing Magnetic Field. *Small* **2018,** *14*, 1800722.
3. Abbott JJ, Fu HC. Controlling Homogeneous Microrobot Swarms In Vivo Using Rotating Magnetic Dipole Fields. In *Springer Proceedings in Advanced Robotics*; Springer: Cham, Switzerland, 2020; Volume 10, pp. 3–8.
4. Byun D, Choi J, Cha K, Park JO, Park S. Swimming microrobot actuated by two pairs of Helmholtz coils system. *Mechatronics* **2011**, *21*, 357–364.
5. Choi H, Choi J, Jang G, Park JO, Park S. Two-dimensional actuation of a microrobot with a stationary two-pair coil system. *Smart Mater. Struct.* **2009**, *18*, 055007.
6. Firester AH. Design of square Helmholtz coil systems. *Rev. Sci. Instrum.* **1966**, *37*, 1264–1265.
7. Kirschvink JL. Uniform magnetic fields and double‐wrapped coil systems: Improved techniques for the design of bioelectromagnetic experiments. *Bioelectromagnetics* **1992**, *13*, 401–411.
8. Baranova VE, Baranov PF. The Helmholtz coils simulating and improved in COMSOL. In Proceedings of the 2014 Dynamics of Systems, Mechanisms and Machines (Dynamics 2014), Omsk, Russia, 11–13 November 2014; pp. 1–4.
